# Towards a More Simplified Approach for Evaluating Strength of Evidence in Health Technology Assessments

**DOI:** 10.7759/cureus.16528

**Published:** 2021-07-21

**Authors:** Frederick Moore, Betsy H Grunch, Larry E Miller, Michael J Musacchio

**Affiliations:** 1 Orthopedics, Southeast Orthopedic Specialists, Jacksonville, USA; 2 Neurosurgery, The Longstreet Clinic, Gainesville, USA; 3 Clinical Research, Miller Scientific, Johnson City, USA; 4 Neurosurgery, NorthShore Spine Center, Evanston, USA

**Keywords:** effectiveness, health technology assessment, healthcare payer, hta, medical device, safety

## Abstract

It is imperative to thoroughly evaluate the safety, effectiveness, and cost-utility of a new medical device prior to the widespread adoption of the technology. Health technology assessment (HTA) is a systematic evaluation of the benefits and harms of a health technology that aims to inform healthcare policy decisions, improve utilization of cost-effective new technologies, and prevent the adoption of devices with harmful or doubtful value for the health system. Even though dozens of organizations perform HTAs, there is no universally accepted criterion for conducting, reporting, and deriving conclusions from an HTA. Thus, there are considerable discrepancies in the methodologies among HTAs such that the same device with the same underlying clinical evidence is often endorsed by one agency but not another, leading to inconsistencies in healthcare coverage policy decisions. Here, we propose a more simplified and unified approach for summarizing clinical effectiveness and safety outcomes for HTAs. We developed a short, semi-quantitative scoring tool that can be used to provide an overall evaluation of evidence strength in HTAs consisting of five categories: (a) the number of randomized controlled trials (RCTs) that have been performed using the technology, (b) the risk of bias among RCTs, (c) the effect size observed for the key effectiveness outcome, (d) the effect size observed for the key safety outcome, and (e) the generalizability of outcomes observed in RCTs to those observed in real-world clinical use. Utilization of this simplified semi-quantitative framework may simplify the HTA process and improve the consistency of the resulting recommendations.

## Introduction

Innovation in medical device development drives advances in healthcare. New medical devices may target a new clinical indication, improve safety and/or effectiveness compared to the existing standard of care, or offer cost savings to patients or healthcare payers. Prior to the widespread adoption of any new medical device, it is imperative to thoroughly evaluate the safety, effectiveness, and cost-utility of the technology. Health technology assessment (HTA) is a systematic evaluation of the benefits and harms of a health technology that aims to inform healthcare policy decisions, improve the utilization of cost-effective new technologies, and prevent the adoption of devices with harmful or doubtful value for the health system. To provide an evidence-based approach to technology evaluation, researchers undertaking an HTA must specify the policy question in terms of safety, effectiveness, and economic aspects in a manner that can be answered by a systematic evaluation of the scientific evidence. Recommendations provided by HTA organizations are increasingly used to inform coverage policy decisions by healthcare payers. Therefore, it is imperative that the methods underlying an HTA for a particular medical device should be as transparent and reproducible as possible.

## Technical report

Evaluation of health technology assessment criteria and methods

In the absence of a universally recognized HTA process, dozens of third-party organizations perform HTAs that are commissioned by governments, medical device manufacturers, or healthcare payers to guide healthcare policy decision-making. Some of these HTAs include Emergency Care Research Institute, National Institute for Health and Care Excellence, Hayes, and Evidence Street, among others. Interestingly, there is no universally accepted criterion for conducting, reporting, and deriving conclusions from an HTA. Thus, there are considerable discrepancies in the methodologies among HTAs (Table 1). These discrepancies are further magnified when different organizations perform HTAs for the same technology, oftentimes with a vague undefined methodology. Ultimately, the same device with the same underlying clinical evidence is often endorsed by one agency but not another, leading to inconsistencies in healthcare coverage policy decisions. Here, we propose a more simplified and unified approach for summarizing clinical effectiveness and safety outcomes for HTAs.

**Table 1:** Comparison of HTA methods among major HTA organizations. ECRI: Emergency Care Research Institute; HTA: health technology assessment; NICE: National Institute for Health and Care Excellence

HTA body	Methodology	Evidence reporting standard
NICE	Systematic review of clinical evidence and economic assessment; testimonies from patients, healthcare professionals, and commissioners	Possible ratings: Guideline recommendations (I-III); levels of evidence (A-C)
Evidence Street	Assessment of five criteria: Appropriate regulatory approval; sufficient evidence to permit conclusions on health outcomes; improves the net health outcome; improvement must be as beneficial as established alternatives; improvement must be attainable outside the investigational setting	Possible ratings: Yes or No
ECRI	Analysis of systematic reviews; review of product-specific clinical literature; review of ongoing clinical trials via clinicaltrials.gov	Possible ratings: Balance of evidence very favorable; balance of evidence somewhat favorable; balance of evidence inconclusive because of no available evidence, mixed results, or too few data; balance of evidence raises concerns; balance of evidence unfavorable
Hayes	Comprehensive systematic review and critical analysis; evaluation of clinical trial data, safety issues, coverage policies, regulatory approvals, and consensus statements; projection of the potential clinical efficacy and economic impact of the technology	Possible ratings: Grade A: Established benefit; Grade B: Some proven benefit; Grade C: Potential but unproven benefit; Grade D1: No proven benefit; Grade D2: Insufficient evidence

Simplified approach for reporting rapid health technology assessment

We developed a short, semi-quantitative scoring tool that can be used to provide an overall evaluation of evidence strength in HTAs (Table 2). The tool consists of five categories including (1) the number of randomized controlled trials (RCTs) that have been performed using the technology, (2) the risk of bias among RCTs, (3) the effect size observed for the key effectiveness outcome, (4) the effect size observed for the key safety outcome, and (5) the generalizability of outcomes observed in RCTs to those observed in real-world clinical use. Each category is scored as +1, 0, or -1, with higher scores representing more favorable evidence and lower scores representing more unfavorable evidence. The scores from each of the five categories are then summed to provide a final score. The final scoring is on a +5 to -5 scale, where higher scores represent evidence with stronger efficacy and safety signals observed in RCTs and in real-world use.

**Table 2:** Proposal for a simplified HTA grading system. HR: hazard ratio; HTA: health technology assessment; MCID: minimal clinically important difference; RCT: randomized trial; RR: risk ratio

Characteristic	1 point	0 point	-1 point
Number of RCTs	2 or more	1	0
Risk of bias among RCTs	Low	Moderate	High, uncertain, or no available RCTs
Effectiveness (MCID units)	1.0 or higher	0.5 to 0.99	Less than 0.5, or uncertain
Safety	Considerably safer than comparator RR or HR <1 (p < 0.05)	Safety similar to comparator. No statistical group differences (p > 0.05)	Considerably more harmful than comparator RR or HR >1 (p < 0.05)
Comparability of outcomes in RCTs to real-world evidence	Highly consistent	Somewhat consistent	Highly inconsistent, or uncertain

Category 1: Number of Randomized Controlled Trials

RCTs represent the highest level of clinical evidence because they are designed to be unbiased and with less risk of systematic design limitations. Further, because clinical results can vary widely from one study to another, data derived from multiple studies provide more reliable evidence than from single studies. Therefore, for purposes of scoring category 1 of the HTA scoring tool, medical devices that have been evaluated in at least two RCTs receive a score of +1, medical devices that have been evaluated in a single RCT receive a score of 0, and medical devices for which no RCTs have been performed receive a score of -1. Importantly, scores are assigned regardless of the number of available noncontrolled studies as evidence derived from these sources is prone to multiple sources of bias.

Category 2: Risk of Bias Among Randomized Controlled Trials

The methods by which RCTs are performed are prone to bias from various sources such as randomization methodology, failure of allocation sequence concealment, baseline imbalances in patient characteristics, and substantial missing outcome data, among others. Given the risk of systematic errors in RCT results due to such biases, the risk of bias appraisal is now a standard reporting item for meta-analyses [1]. As such, category 2 of the scoring tool assigns the highest score of +1 where RCTs have an overall low risk of bias as evaluated by a systematic literature review, a score of 0 where the risk of bias is deemed moderate, and a score of -1 when the risk of bias is high or uncertain, or when no RCTs are available.

Category 3: Effectiveness

Category 3 utilizes the results of a meta-analysis of the key effectiveness outcome to assign points. To facilitate clinical interpretation of the meta-analysis results for endpoints measured on a continuous scale, the treatment effects are transformed to standardized minimal clinically important difference (MCID) units [2,3], where the standardized MCID for the primary outcome is calculated as the treatment benefit divided by the MCID. As an example, consider a medical device in which the treatment benefit compared to a control group is 15 points on a 0-100 visual analog pain scale. If the established MCID on this pain scale is 20 points, then the standardized MCID is calculated as 15 divided by 20, or 0.75. For reference, improvements greater than 1 MCID unit (assigned a score of +1) indicate that many patients may gain important benefits from treatment, improvements between 0.5 and 1 MCID units (assigned a score of 0) suggest that the treatment may benefit an appreciable number of patients, and an improvement of less than 0.5 MCID units (assigned a score of -1) suggests that it is unlikely that an appreciable number of patients will show a clinically important benefit [2,3]. Outcomes measured on a binary scale are typically reported as a hazard ratio, an odds ratio, or a risk ratio in a meta-analysis, where values less than 1 with a p-value of <0.05 favor the device and values greater than 1 with a p-value of <0.05 favor the control. Meta-analysis results favoring the device would be assigned +1 point on the scoring tool, results demonstrating no statistical differences between groups would be assigned 0 point, and results favoring the control would be assigned -1 point.

Category 4: Safety

Category 4 evaluates the key safety outcome and is scored identically as effectiveness, depending on whether the outcome is scored on a continuous or binary scale.

Category 5: Comparability of Outcomes in Randomized Controlled Trials to Real-World Evidence

The generalizability of the RCT data can be compared to data derived from nonrandomized studies to determine whether similar results are attained. This element of the scoring tool is not easily determined with statistical analyses. Instead, it is recommended to qualitatively evaluate the strength and direction of the treatment effect for effectiveness and safety outcomes. If the results from RCTs and non-RCTs both suggest similar outcomes, then 1 point is assigned. If the results of this analysis are indeterminate, then 0 point is assigned. If no RCTs have been performed or if the results from RCTs are distinctly different from those derived from non-RCTs, then -1 point is assigned.

Example of simplified approaches for reporting health technology assessments

The Barricaid Annular Closure Device (Intrinsic Therapeutics, Woburn, MA) is a Food and Drug Administration-approved intervertebral biomechanical device that consists of a woven polyethylene terephthalate (PET) flexible fabric component that attaches to a titanium alloy (Ti-6Al-4V ELI) intravertebral bone anchor [4]. The bone anchor component is used to secure the device to one of the adjacent vertebral bodies and ensure the correct positioning of the flexible fabric component in front of an annular defect. The occlusion component consists of a flexible polymer that is designed to prevent reherniation by physically blocking the annulus at the postsurgery defect to maintain hydrostatic pressure inside the nucleus pulposus, and containing a platinum-iridium radiopaque marker to permit radiographic visualization. The device is implanted following lumbar discectomy and is intended to physically occlude large annular defects and, consequently, lower the risk of reherniation.

In a systematic review with meta-analysis of RCTs and non-RCTs of this device [5], it was determined that two RCTs had been performed [6,7]; the risk of bias in the RCTs was graded as moderate; the risk ratio for the key effectiveness endpoint of symptomatic reherniation was 0.47 (p < 0.01) favoring the device versus controls; the risk ratio for the key safety endpoint of device- and procedure-related serious adverse events was 0.57 (p = 0.009) favoring the device versus controls [6]; and clinically meaningful and statistically significant reductions in symptomatic reherniation, low reoperation rates, and a favorable safety profile were demonstrated in the meta-analysis comparing RCTs and nonrandomized studies. Using the semi-quantitative HTA scoring model, the underlying evidence for the Barricaid device would be scored as +1 point for Category 1, 0 point for category 2, and +1 point for Category 3, 4, and 5, yielding a total score of 4 out of 5 (Table 3).

**Table 3:** Application of the simplified HTA approach to existing evidence for an annular closure device for the prevention of lumbar disc reherniation. *Primary evidence provided by a systematic review with meta-analysis of controlled and noncontrolled studies [5]. HTA: health technology assessment; RCT: randomized trial

Characteristic	Assigned points	Maximum possible points	Rationale*
Number of RCTs	1	1	Two RCTs have been performed [6,7]
Risk of bias among RCTs	0	1	Risk of bias in RCTs was graded as moderate in a previous meta-analysis [5]
Effectiveness (MCID units)	1	1	Risk ratio of 0.47, signifying a 53% relative reduction in symptomatic reherniation and reoperation risk noted in patients treated with Barricaid vs. controls [5]
Safety	1	1	Risk ratio of 0.57 (p = 0.009), indicating a 43% relative reduction in device- and procedure-related serious adverse events with Barricaid vs. controls [6]
Comparability of outcomes in RCTs to real-world evidence	1	1	Clinically meaningful and statistically significant reduction in symptomatic reherniation and reoperation rates was demonstrated in a meta-analysis of RCTs and nonrandomized studies [5]
Total score	4	5	

## Discussion

The development and adoption of health technology are increasingly influenced by regulators, healthcare payers, clinicians, patients, and government leaders who demand rigorous scientific evidence to support policy decisions. The proliferation of HTA methods continues to evolve and is becoming increasingly diverse as many organizations now have their own HTA units. Given the vast differences in HTA methodologies, there is a need for a simplified HTA scoring tool that can be completed quickly and reliably.

In recent years, the demand for HTA by healthcare decision-makers has increasingly involved requests for faster responses to help provide timely decisions. This has led to the development of rapid HTAs [8]. While rapid HTAs provide less comprehensive information relative to a full HTA, there is less need for resources, and completion time is much faster [9]. However, there is no current consensus regarding methodology or guidance for rapid HTAs [10]. If HTAs are not conducted and reported in a transparent manner, it is impossible to determine the validity and usefulness of the recommendations. We, therefore, developed the initial framework of a simplified scoring tool by which to grade the strength of scientific evidence for purposes of rapid HTA evaluation.

There are several advantages of using this approach. First, the framework utilizes a simple 11-point scoring scale which should enhance interpretability as a positive score suggests favorable overall evidence and a negative score suggests unfavorable overall evidence. Second, several of the scoring assessments are based on quantitative information such as the number of RCTs, risk ratios, and other statistical metrics, which should facilitate high interrater scoring consistency. Finally, a simplified, semi-quantitative scoring model might help to minimize one of the primary limitations of HTA, namely, the issue whereby HTA bodies that evaluate the same set of evidence may derive vastly different conclusions and recommendations. To the extent that dozens of HTA bodies currently exist and all use different assessment criteria, a paradigm shift towards more simplified reporting and transparent decision-making based on quantitative summary statistics may remove bias and subjectivity from HTA decisions.

There are also several limitations of our proposed approach. This abbreviated methodology may not capture the full complexity of the underlying clinical data, and refinements may be needed to adequately handle special circumstances that may be specific to certain technologies. Numerous additional factors weigh into a comprehensive HTA appraisal such as the regulatory status of a product, cost-effectiveness of the data, existing societal guidelines, funding sources, and study sample size, among others. These additional factors are certainly intended to be added as supportive information, which would be considered in conjunction with the grading from the 11-point scoring scale. Additionally, the interrater agreement of our semi-quantitative scoring tool remains unclear. However, in theory, it may be higher than with current approaches that use disparate and mainly qualitative assessment methods. Finally, this simplified model does not consider the cost-utility of medical technologies. A number of HTA organizations attempt to formulate recommendations based on the collective effectiveness, safety, and cost-utility of the evidence. We argue that safety, effectiveness, and cost-utility should be evaluated in separate stages. That is, if the risk-to-benefit balance of a medical technology is deemed unfavorable based on effectiveness/safety data, it would be futile to additionally perform cost-utility studies. We, therefore, propose a simplified approach for the assessment of effectiveness/safety in this paper. Based on the risk-to-benefit profile established at this stage, subsequent cost-utility analyses would be warranted, which are beyond the scope of the current paper.

## Conclusions

Recommendations provided by HTA organizations are increasingly used to inform coverage policy decisions by healthcare payers. However, the same health technology is often assessed by different HTA agencies, often deriving different recommendations due to differences in HTA methodologies. A simplification of these methods, whereby a semi-quantitative effectiveness/safety scoring tool is used, may help to simplify and standardize the HTA process.
